# Análisis crítico de la conceptualización del trabajo en personas mayores en la época contemporánea

**DOI:** 10.1590/0102-311XES094925

**Published:** 2026-02-06

**Authors:** José Rosario González-Ulloa, Adriana Elizabeth Morales-Sánchez, Cecilia Andrea Ordoñez-Hernández, Georgina Vega-Fregoso, Ricardo Fletes-Corona

**Affiliations:** 1 Universidad de Guadalajara, Guadalajara, México.; 2 Universidad Libre, Cali, Colombia.

**Keywords:** Jubilación, Personas Mayores, Trabajo, Teoría Social, Retirement, Aged, Work, Social Theory, Aposentadoria, Idoso, Trabalho, Teoria Social

## Abstract

El presente ensayo crítico analiza las conceptualizaciones que se han realizado sobre el trabajo en la vejez entre 2000 y 2025, desde la visión occidental. Ante el surgimiento de nuevas relaciones laborales en este mundo globalizado, se vuelve imperante, ajustar o construir definiciones conceptuales del trabajo que permitan visualizar de manera integral sus implicaciones en las personas mayores. El objetivo de este ensayo fue argumentar cómo la conceptualización unidimensional del trabajo disminuye la capacidad de explicar las nuevas dinámicas en el trabajo de personas mayores. En cuanto a la metodología utilizada, esta investigación siguió el esquema de ensayo crítico, utilizando una lógica inductiva, partiendo de lo particular a lo general, bajo el modelo argumentativo de Toulmin. Se halló que las conceptualizaciones sobre el trabajo en la vejez se han concentrado en los niveles micro y macrosocial. A nivel microsocial se identificaron los conceptos de envejecimiento productivo, identidad laboral, terapia ocupacional y adicción al trabajo (*workaholism*). Estos conceptos están relacionados con experiencias, transiciones de roles, ventajas y desventajas que perciben las personas mayores trabajadoras. En el nivel macrosocial se encontraron los conceptos de trabajo no clásico, trabajo decente y división sexual del trabajo, los cuales reconocen la influencia de los aspectos económicos, políticos y culturales en las relaciones laborales en la vejez. En conclusión, las conceptualizaciones que se han construido hasta el momento todavía no logran consolidar una teoría explicativa que permita establecer una definición del trabajo en la vejez.

## Abstract (en)

This critical essay analyzes conceptualizations of work in aged individuals between 2000 and 2025 from a Western perspective. Given the emergence of new labor relations in this globalized world, it is imperative to adjust or construct conceptual definitions of work that allow for a comprehensive view of its implications for older adults. The objective of this essay was to argue how the one-dimensional conceptualization of work reduces the ability to explain the new dynamics in the work of older people. In terms of the methodology used, this research followed the critical essay format, using inductive logic, starting from the specific to the general, under Toulmin’s argumentative model. We found that conceptualizations of work in old age have focused on the micro and macrosocial levels. At the microsocial level, the concepts of productive aging, work identity, occupational therapy, and workaholism were identified. These concepts are related to experiences, role transitions, advantages, and disadvantages perceived by older workers. At the macrosocial level, the concepts of non-traditional work, fair work, and the gender division of labor were identified, which recognize the influence of economic, political, and cultural aspects on labor relations in old age. In conclusion, the conceptualizations that have been developed so far have not yet succeeded in consolidating an explanatory theory that would allow us to establish a definition of work in old age.

## Abstract (pt)

Este ensaio crítico analisa as conceituações feitas sobre o trabalho em idosos entre 2000 e 2025, a partir de uma visão ocidental. Diante do surgimento de novas relações trabalhistas no mundo globalizado, torna-se imprescindível ajustar ou construir definições conceituais do trabalho que permitam observar de forma integral suas implicações para os idosos. O objetivo deste ensaio foi argumentar como a conceituação unidimensional do trabalho diminui a capacidade de explicar as novas dinâmicas no trabalho dos idosos. Quanto à metodologia utilizada, esta pesquisa seguiu o esquema do ensaio crítico, utilizando uma lógica indutiva, partindo do particular para o geral, sob o modelo argumentativo de Toulmin. Verificou-se que as conceituações sobre o trabalho na velhice se concentraram nos níveis micro e macrosocial. No nível microsocial, foram identificados os conceitos de envelhecimento produtivo, identidade profissional, terapia ocupacional e vício em trabalho (*workaholism*). Esses conceitos estão relacionados com experiências, transições de papéis, vantagens e desvantagens percebidas pelos idosos que trabalham. No nível macrosocial, foram encontrados os conceitos de trabalho não clássico, trabalho digno e divisão sexual do trabalho, que reconhecem a influência dos aspectos econômicos, políticos e culturais nas relações laborais na velhice. Em conclusão, as conceituações construídas até o momento ainda não conseguem consolidar uma teoria explicativa que permita estabelecer uma definição para o trabalho na velhice.

## Introducción

El trabajo en la vejez es un tema que tomó relevancia a mediados del siglo pasado, puesto que, las condiciones en que las personas mayores se incorporan al trabajo se han modificado y se ha vuelto cada vez más difícil ubicar las fronteras entre el trabajo y el no trabajo, debido a la diversidad y flexibilidad que han adoptado las relaciones laborales en esta etapa vital ^1^.

Este fenómeno adquiere características específicas que requieren ser resignificadas, ante las realidades en las que viven las personas mayores que necesitan seguir trabajando. Para ello, es importante identificar cómo se han problematizado desde la visión occidental las diferencias contextuales en las que están inmersas las personas mayores que viven en países del norte global y del sur global, así como, la forma en que se han analizado las trayectorias laborales, los significados que le atribuyen a esta actividad ^2^, y la relevancia que tiene esta problemática para la salud pública.

Para abordar el trabajo en la vejez se han utilizado enfoques micro y macrosociales que proponen una mirada longitudinal que abarca dimensiones biográficas e históricas. En la dimensión biográfica se toman en cuenta las experiencias laborales, las transiciones de roles y la sumatoria de ventajas y desventajas sociales; mientras que, en la dimensión histórica, se reconoce el impacto de hitos que marcaron una determinada etapa de la trayectoria laboral ^3^.

Por lo tanto, los niveles de participación laboral de las personas mayores varían según el país, los grupos de edad y el sexo, así como, el tipo de ocupación y el nivel de ingresos ^4^. Ahora bien, los trabajadores de más edad afrontan dificultades en la contratación, en el acceso a oportunidades de capacitación, en la transición profesional y en la posibilidad de realizar tareas adaptadas a sus capacidades físicas e intelectuales ^5^.

Así que, mediante este ensayo crítico, se busca analizar cómo la conceptualización del trabajo en la vejez se ha construido entre dos niveles de interacción: microsocial y macrosocial. Se propone argumentar cómo esta conceptualización diferenciada ha disminuido la capacidad de explicar las nuevas dinámicas que surgen el trabajo durante la vejez y poder responder a la pregunta: ¿cómo reconceptualizar el trabajo en la vejez para ampliar su comprensión?

Para lograrlo, se desarrolló el esquema de un ensayo crítico, que consta de introducción, desarrollo y conclusión. Se establecieron dos categorías temáticas y se presentaron las conceptualizaciones sobre el trabajo en la vejez utilizando una lógica inductiva, partiendo de lo particular a lo general, bajo el modelo argumentativo de Toulmin ^6^.

**Figura 1:**
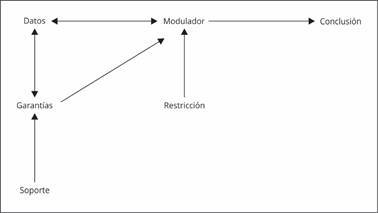
Esquema del modelo argumentativo de Toulmin.

Este modelo tiene una estructura que permite plantear ideas que son puestas a debate, se utilizan datos para respaldarlas (Datos), a continuación se introduce una justificación que sirve de enlace entre los datos y las conclusiones (Garantías), mientras que, se consideran variaciones (Modulador) o restricciones (Refutación potencial) que pueden estar presentes en el fenómeno investigado. Además, se fortalece la premisa con elementos teóricos conceptuales (Soporte), lo cual, permite construir un argumento sólido y conclusiones adecuadas ^6^ (Figura 1).

Fuente: elaboración propia, basada en Marafioti ^6^.

## Conceptualización a nivel microsocial del trabajo en la vejez

A nivel microsocial, el trabajo en la vejez ha sido abordado desde tres dimensiones principales: (a) experiencias laborales; (b) transiciones de roles; y (c) ventajas y desventajas individuales. Estas dimensiones son acompañadas de teorías, métodos, metodologías y conceptos acuñados por cada uno de esos marcos de referencia (Cuadro 1). A continuación, revisaremos los elementos que integran cada una de esas dimensiones.

**Cuadro 1:** Abordajes del trabajo en la vejez desde el enfoque microsocial.

DIMENSIÓN	TEORÍAS	CONCEPTOS
Experiencias laborales	Teoría de la continuidad (Atchley ^41^)	Continuidad laboral. Evolución que mantiene conexión con el pasado de las personas y permite una maduración gradual de las capacidades de adaptación (Atchley ^41^)
	Curso de vida (Giele & Elder ^42^)	Envejecimiento productivo. Capacidad de un individuo o población para servir en la fuerza de trabajo remunerada, en actividades de voluntariado, ayudar en la familia y mantenerse independiente para contribuir al desarrollo de la sociedad (Mirelles ^9^)
	Envejecimiento activo (Organización Mundial de la Salud ^11^)	Envejecimiento activo. Es el proceso de optimización de las oportunidades de salud, participación y seguridad con el fin de mejorar la calidad de vida a medida que las personas envejecen (Organización Mundial de la Salud ^11^)
Transiciones de roles	Construcciones identitarias en el trabajo (Afonso ^13^)	Identidad laboral. Construcción personal de la finalidad y del sentido del trabajo que son legitimadas como significados colectivos (Afonso ^13^)
Ventajas y desventajas sociales	Teoría de la autodeterminación (Deci & Ryan ^43^)	Terapia ocupacional. Permite a los trabajadores mayores que elijan ocupaciones significativas que faciliten la transición del trabajo a la jubilación (Eagers et al. ^17^). *Workaholism.* Constructos que caracteriza las cualidades del trabajo arduo, largas jornadas laborales que excede las exigencias del puesto y una fuerte dedicación al trabajo, asociado a emociones desagradables (Malinowska et al. ^19^)

Fuente: elaboración propia.

### Experiencias laborales en la vejez

A partir de la década de los 1960 se experimentó un auge en la investigación sobre el trabajo en la vejez en Norteamérica. A partir de las ideas de las teorías sociológicas del envejecimiento de primera generación (teoría la desvinculación, teoría de la actividad y teoría de la subcultura), Robert Atchley desarrolló la Teoría de la Continuidad en la que estudia los procesos de adaptación que ocurren en la jubilación, en ella, plantea que las personas mayores buscan mantener sus rutinas y autoimagen positiva a través de actividades significativas ^7^.

Si bien es cierto que, quienes tienen experiencias positivas en sus carreras profesionales tienden a continuar en el mismo empleo, hay otras personas que tienen rupturas abruptas de su vida laboral y no pueden mantener esa continuidad. Por esa razón, se critica la teoría de la continuidad, ya que da un mayor énfasis a la perspectiva individual y relacional con los otros, pero, desestima la influencia de los elementos del contexto sociocultural en los que está inmerso el sujeto ^8^.

Para elaborar un análisis más profundo de las experiencias del trabajo en la vejez en Latinoamérica, Mirelles ^9^ utilizó el concepto de envejecimiento productivo, basándose en la Teoría de Curso de Vida, reconociendo cómo las trayectorias laborales influyen en los significados que las personas le confieren al trabajo, así como, sus valores asociados. En este enfoque se incluye el análisis de las diferencias de género, tanto en las trayectorias laborales, como en los valores que hombres y mujeres le atribuyen al trabajo en la vejez.

Por su parte, Wadell et al. ^10^ hacen una crítica al modelo de envejecimiento exitoso de Rowe y Khan, pues no consideran que la ausencia de enfermedad, mantener capacidad física y funcionalidad cognitiva, son los elementos necesarios para lograr un envejecimiento exitoso. Sin embargo, fue a partir de esta concepción utilitarista, que se vislumbraron las primeras políticas públicas en torno al trabajo en la vejez y para el desarrollo del concepto de envejecimiento activo que adoptó la Organización Mundial de la Salud (OMS) en la década de 1990.

Este marco político de la OMS surge como respuesta ante la transición demográfica que se vive a nivel global en la actualidad. A nivel individual busca potenciar la participación continua de las personas mayores en las cuestiones sociales, económicas, culturales, espirituales y cívicas, para mantener su autonomía e independencia. Para ello, analiza los determinantes sociales, conductuales, personales, económicos, entorno físico y sanidad, con dos ejes transversales: el sexo y la cultura ^11^.

### Transiciones de roles de personas mayores trabajadoras

En la búsqueda de comprender las transiciones a lo largo de la vida, la Teoría de Roles de Linton propone que los sujetos están personificando roles asociados a expectativas sociales que pueden afectar la imagen auto percibida y también producir estereotipos hacia las personas que no cumplen con esas expectativas ^12^. A partir de este enfoque se puede analizar el concepto de identidad laboral.

La identidad laboral se construye en las dimensiones: vocacional, relacionada al ser; ocupacional y profesional, relacionada al hacer; organizacional, vinculada al espacio de trabajo; de carrera, asociada a la trayectoria laboral ^13^. Una condición importante en la identidad laboral es la edad, ya que tiene una influencia relevante en la decisión de transitar del trabajo de tiempo completo a trabajo parcial o retiro total. En América Latina, las tasas de participación del grupo de 60 a 64 años (50,2%) fueron las más elevadas en 2020, mostrando una disminución significativa en los grupos de mayor edad, de 70 a 74 años (25,3%) y de 75 a 79 años (18,7%), aunque las tasas de participación de las mujeres fueron menores (33,3% de 60 a 64 años) que la de los hombres (69,4% de 60 a 64 años) y aumenta la brecha con la edad (11% contra 28,4% en el grupo de 75 a 79 años) ^14^.

En México, gran parte de los adultos mayores laboran, tanto a tiempo parcial como a tiempo completo, con bajos salarios, precarización laboral, incertidumbre y la falta de seguridad social. Pese a la edad avanzada y las condiciones biopsicosociales en las que se encuentran, tienen la necesidad de emplearse, ya sea por falta de pensión o por pensiones bajas, que no les permite mantener una vida digna ^15^.

### Ventajas y desventajas del trabajo en la vejez

Una de las principales motivaciones para continuar trabajando en la vejez es el cuidado de la salud. Utilizando el concepto de ocupaciones productivas con el enfoque de terapia ocupacional se ha buscado potenciar las habilidades de las personas mayores para que puedan satisfacer deseos y mantener una rutina en su vida cotidiana, lo cual, tiene implicaciones económicas y contribuye a conservar su identidad y valores ^16^.

Considerando los beneficios del trabajo como una terapia ocupacional, Eagers utilizó el Modelo de Ocupación Humana (MOHO) en participantes de 58 a 75 años en Australia y observó que el trabajo en la vejez ofrece estructura y objetivo a cada día, permite un balance entre el trabajo y el descanso, otorga identidad y permite la interacción con otras personas ^17^.

Aunque, los resultados de una revisión sistemática hecha en 2021 muestran que los mayores beneficios del trabajo en la vejez solo están presentes en el grupo de 60 a 70 años, se destaca que el tipo de empleo y las condiciones de trabajo son factores que contribuyen a la ampliación de desigualdades en trabajadores mayores que presentan bajos niveles de salud y educación en países del sur global, donde hay bajo reconocimiento y altas exigencias laborales ^18^.

A pesar de los beneficios que otorga el trabajo en la vejez, estos deben ser tomados con cautela. Que las personas mayores continúen trabajando conlleva riesgos de accidentes laborales, puede producir explotación, estrés, desgaste emocional y físico. En occidente se ha comenzado a analizar el concepto de adicción al trabajo (*workaholism*) y se ha encontrado que los adictos al trabajo continúan trabajando porque desean cumplir con sus propias expectativas y las de los demás o como estrategia para evitar emociones desagradables ^19^.

Por lo tanto, es importante reconocer que mediante los conceptos mencionados sobre el trabajo en la vejez a nivel microsocial, se hace un análisis de la capacidad de adaptación a los cambios que se producen en las condiciones de trabajo, en la capacidad funcional y a los motivos para continuar trabajando, sin embargo, ha predominado la visión utilitarista del trabajo en personas mayores, invisibilizando desigualdades estructurales, diferencias de género y la desarticulación de políticas públicas para este sector de la población.

Así pues, desde la salud pública es necesario fortalecer los procesos de investigación sobre el trabajo en la vejez, ir más allá del análisis descriptivo de los aspectos positivos y negativos en la salud de las personas mayores. Hay que reconocer que existen procesos de envejecimiento heterogéneos en la población en los que intervienen elementos económicos, políticos y culturales, por lo cual, es primordial generar procesos de investigación participativa.

## Conceptualización a nivel macrosocial del trabajo en la vejez

A nivel macrosocial, los conceptos se desarrollan en tres dimensiones: (a) económica; (b) política; y (c) cultural. El abordaje teórico de la dimensión económica se realiza mediante el configuracionismo. La dimensión política considera como base fundamental los derechos humanos y la dimensión cultural se analiza con una perspectiva de género (Cuadro 2).

**Cuadro 2:** Abordajes del trabajo en la vejez desde el enfoque macrosocial.

DIMENSIÓN	TEORÍA	CONCEPTO
Económica	Configuracionismo (De la Garza ^20^)	Trabajo no clásico. Se trata de un trabajo que puede implicar interacciones, generación de símbolos como parte substancial del producto, y el trabajo del cliente, así como la evolución subjetiva entre el demandante del trabajo y el oferente (De la Garza ^20^)
Envejecimiento activo (Organización Mundial de la Salud ^11^)	Economía plateada. Actividades económicas relacionadas con la producción, el consumo y el comercio de bienes y servicios relevantes para las personas mayores, tanto públicas como privadas (Casas ^23^)
Política	Derechos humanos (Naciones Unidas ^44^)	Trabajo decente. El trabajo decente sintetiza las aspiraciones de las personas durante su vida laboral. Significa la oportunidad de acceder a un empleo productivo que genere un ingreso justo, la seguridad en el lugar de trabajo y la protección social para todos, mejores perspectivas de desarrollo personal e integración social, libertad para que los individuos expresen sus opiniones, se organicen y participen en las decisiones que afectan sus vidas, y la igualdad de oportunidades y trato para todos, mujeres y hombres (Somavía ^37^).
Cultural	Teoría feminista (Scott ^45^)	División sexual del trabajo. Una división sexual del trabajo determina la actividad de la gente, sus propósitos, deseos y sueños de acuerdo con su sexo biológico, está en la base del patriarcado y del capitalismo: divide a los hombres y a las mujeres y los coloca en sus respectivos papeles sexuales jerarquizados además de estructurar sus deberes en relación con el dominio específico de la familia dentro de la economía (Eisenstein ^46^)

Fuente: elaboración propia.

### Dimensión económica. Trabajo no clásico

El concepto de trabajo no clásico surge del paradigma crítico, es utilizado para dar cuenta de las actividades productivas que parten de relaciones de trabajo ampliadas, flexibles, simbólicas e inmateriales que se han consolidado con la globalización ^20^. El trabajo en la vejez en los países del sur global, generalmente, se desarrolla en actividades laborales periféricas, como pueden ser taxistas, conductores en plataformas, voluntariado en cadenas de supermercado, comercio informal, entre otros.

Además, el trabajo en la vejez tiene características diferenciales al trabajo que se realiza en las etapas anteriores de la vida, porque ya no está asociado su valor como mercancía, sino a la subjetividad que se configura mediante la interacción de códigos estéticos, morales y cognitivos que se producen durante las relaciones de trabajo ^20^.

En el panorama global, también están presentes las nuevas tecnologías de inteligencia artificial que tienen el potencial de: “*transformar las empresas, los sectores productivos, los mercados de trabajo y a la sociedad en general*” ^21^ (p. 6). Sin embargo, las personas mayores pueden ser más vulnerables a los impactos negativos de la automatización, la digitalización y la falta de regulación de los mercados laborales.

### Economía plateada

Ante los cambios que ha traído consigo el modelo económico neoliberal adoptado en gran parte del mundo, se han transformado los estándares del trabajo clásico asociados al modelo capitalista impulsado en la Revolución Industrial ^21^. En este contexto de globalización surge el concepto de economía plateada, vinculado al cambio demográfico, pero, centrado en las demandas y necesidades de las personas mayores ^22^.

Si bien es cierto que este concepto busca darle una noción positiva a la participación de las personas mayores en el mercado laboral, no se puede dejar de lado que su población objetivo, son personas entre 50 y 70 años, que todavía gozan de buena salud y que además son potenciales consumidores de bienes y servicios, por lo tanto, bajo esa lógica pasan de ser agentes activos a ser consumidores pasivos, además, se excluye a las personas mayores en situación de pobreza o quienes son longevas ^23^.

### Dimensión política. Trabajo decente

La *Declaración Universal de los Derechos Humanos* en 1948 en Asamblea General de las Naciones Unidas indica que: “*todos tienen derecho a trabajar*” en condiciones favorables que les permitan a las personas satisfacer sus necesidades y alcanzar una calidad de vida digna. Basado en esta premisa, la Organización Internacional del Trabajo (OIT) construye el concepto de trabajo decente ^24^.

Más que una definición teórica, el trabajo decente es una visión instrumental que busca establecer indicadores que permitan verificar que existan empleos suficientes, remunerados y con condiciones de seguridad para desarrollarse. Además, se integran la seguridad social y la protección jurídica como elementos complementarios para proteger de las inequidades que pueden presentar grupos vulnerables ^25^.

Desafortunadamente, el trabajo en la vejez, lejos de ser un derecho, parece haberse convertido en un castigo. Actualmente, existe la tendencia en los países con mayor nivel de envejecimiento poblacional de buscar incrementar la edad de jubilación. Por ejemplo, Japón la incrementó de 60 a 65 años en 2005, en Singapur pasará de 65 a 70 años en 2030, en Dinamarca el aumento de un año fue acordado en 2015 y pasará en 2030 de 67 a 68 años, y en Países Bajos se aprobó que la edad pasará de 64 a 66 años en 2024 ^26^.

Ahora bien, las condiciones son distintas para las personas que trabajan en la informalidad y no logran acceder a la seguridad social. En América Latina las tasas de informalidad en personas mayores de 60 años y más alcanzó el 67,6% en 2022, además, se observó que muchos casos las personas mayores dependen económicamente de estos trabajos para complementar ingresos, apoyar a sus familias o para cubrir sus propias necesidades básicas, lo que podría perpetuar la informalidad en este grupo al no poder retirarse completamente ^27^.

De tal manera, que el concepto de trabajo decente está perfilado hacia el trabajo formal y para los trabajadores jóvenes y adultos que lo realizan, relegando a los adultos mayores trabajadores informales y quienes realizan trabajos no clásicos que se encuentran en los límites entre la formalidad y la informalidad.

### Dimensión cultural. División sexual del trabajo

El género es una categoría que atraviesa las relaciones laborales y evidencia como las diferencias de sexo producen procesos de dominación; cómo se desarrollan las relaciones de poder y símbolos culturales y cómo permean en las estructuras del sistema patriarcal y se refuerzan a través del capitalismo ^28^.

La complejidad de establecer un análisis de la división sexual del trabajo en la vejez es que existe una acumulación de desigualdades durante la trayectoria laboral de hombres y mujeres que se ven reforzadas por el sistema patriarcal y las políticas neoliberales que las justifican en el ámbito público y en el privado ^29^.

En el caso de la mayoría de las mujeres trabajadoras mayores latinoamericanas, están expuestas a un mayor riesgo de pobreza porque generalmente los montos de pensiones que reciben son más bajos, perciben sueldos más bajos que los hombres; durante sus trayectorias laborales ocurren interrupciones y discriminaciones laborales, además, tienen una doble jornada realizando actividades de cuidado en la família ^30^.

En cambio, durante la vejez los hombres experimentan transformaciones en los roles y privilegios que pudieron tener en otras etapas de la vida. Quienes dejan de trabajar, pierden los recursos de poder y control que les proporcionaba el rol de ser proveedores, además, se integran al ámbito del hogar, donde en ocasiones no son bien recibidos, porque esos espacios domésticos están culturalmente asociados a roles femeninos ^31^.

Por ello, la inclusión de la mujer en el ámbito laboral ha traído consigo cambios en las configuraciones del espacio público y del espacio privado, convirtiendo una crisis en la imagen del rol de la masculinidad moderna. Esa transformación ocurrida en las últimas dos décadas ha aumentado el poder de negociación de las mujeres frente a los hombres, y ha disminuido la capacidad de dominio que ostentaban los hombres en el pasado.

Finalmente, el abordaje conceptual que se ha realizado del trabajo en la vejez a nivel macrosocial refleja la complejidad del contexto en el que están inmersas las relaciones laborales en la era de la globalización. A pesar de que en las últimas décadas la salud pública ha integrado en sus procesos de análisis los determinantes sociales de la salud, todavía no se ha logrado consolidar un puente teórico-metodológico que enlace la parte microsocial con la macrosocial.

## Discusión sobre la conceptualización del trabajo en la vejez

En el presente ensayo se realizó un análisis crítico de la conceptualización del trabajo en la vejez en el siglo XXI, principalmente desde la visión occidental. Se reconocieron dos dimensiones teórico-metodológicas micro y macrosocial bajo las cuales se ha definido el trabajo en la vejez. La primera dimensión incorpora los significados, expectativas, experiencias y valores asociados al trabajo, así como, las motivaciones de las personas mayores para seguir trabajando en la vejez. La segunda reconoce que las condiciones contextuales que dan vida a este fenómeno social también atraviesan las relaciones laborales de hombres y mujeres dentro de un marco político en un mercado laboral globalizado.

Si bien es cierto que los valores y significados positivos que la mayoría de las personas mayores atribuyen al trabajo son relevantes, es importante dar cuenta de la heterogeneidad de las trayectorias de vida, para no caer en una visión simplista que limite la comprensión de la complejidad de este fenómeno social.

Por ello, se considera adecuado utilizar el enfoque de curso de vida en el concepto de envejecimiento productivo, porque como lo mencionan Blanco & Pacheco ^32^, los significados se construyen a partir de experiencias individuales, se refuerzan mediante las interacciones con los demás que se ven marcadas transiciones en los procesos históricos vividos.

Por su parte, el concepto de envejecimiento productivo fue utilizado por Oddone & Chernobilsky ^33^ para dar un sentido utilitarista al trabajo en la vejez, considerando los beneficios que produce para el individuo, para la comunidad y para la sociedad, principalmente en el aspecto económico.

Sin embargo, utilizando esta visión para conceptualizar el trabajo en la vejez, se corre el riesgo de pensar que todas las personas mayores deberían de seguir trabajando para gozar de ese derecho. Como lo señala Fuentes-García ^34^, también se deben considerar las condiciones de vulnerabilidad laboral y desigualdad social en las que viven personas mayores en Latinoamérica.

Desde la dimensión macrosocial Katz & Wailes ^1^, hacen énfasis en que las relaciones laborales en la era de la globalización han ampliado las posibilidades de realizar actividades productivas y se ha desfigurado la imagen que se tiene del trabajo clásico. Cada vez es más difícil reconocer las fronteras entre el trabajo y el no trabajo, máxime cuando se realiza en la vejez, puesto que, las características de las relaciones laborales en etapa vital se dan bajo perspectivas distintas que en la juventud y la adultez.

Por una parte, la expectativa social es que las personas mayores ya no trabajen, por esa razón, Porcellato et al. ^35^ indican que quienes siguen trabajando están ocupando un espacio que no se tenía contemplado. Además, existe una contradicción en cuanto al derecho a trabajar que tienen estas personas, por ejemplo, en el caso de México, para quienes tienen un trabajo formal, la *Ley Federal del Trabajo* establece una edad para obtener la pensión por cesantía, mientras que en los contratos laborales se acuerdan los años de servicio necesarios para la pensión por vejez ^36^.

Estos límites de edad en ocasiones son el pretexto perfecto para que las empresas u organizaciones se deshagan de los “viejos”. A su vez, cuando se llega a la vejez disminuyen las oportunidades de trabajo, por esa razón, se ven orillados a aceptar trabajos precarios, informales y sin seguridad social o actividades de voluntariado.

Empero, el concepto de trabajo decente que propone la OIT tiene poca aplicación en la realidad en que viven las personas mayores trabajadoras, puesto que se enfoca en la evaluación de las condiciones de trabajo en empleos formales ^37^. De tal manera, que se torna complejo con este concepto dar cuenta de modalidades de trabajo informal o trabajo no clásico, como son: autoempleo, el trabajo creativo y el trabajo inmaterial o voluntariado.

El trabajo decente se afirma en cuatro pilares. El primero es el empleo; el segundo, son los derechos laborales; el tercero, son la seguridad y protección social; y, el cuarto, es la representación y diálogo social. Estos cuatro pilares se refuerzan recíprocamente y contribuyen a la integración social y al desarrollo personal de los trabajadores ^37^.

A la par de las condiciones de vulnerabilidad que viven las personas mayores trabajadoras, se dan las diferencias producidas por cuestión de género. La división sexual de trabajo impuesta por el sistema sexo/género se ha anquilosado en la estructura social y se legitima por las instituciones ^38^. Dando como resultado la intersección de las desigualdades en las trayectorias de vida de las mujeres y esa suma de elementos cobran factura en la vejez ^39^.

Sin embargo, el proceso de producción basado en el género también crea una serie de tensiones y desigualdades para los hombres, porque también ellos son excluidos del sistema productivo cuando están desempleados, tienen trabajos con poco reconocimiento social o no cumplen con los roles y expectativas de “ser hombres” ^40^.

Por ello, para dar una conceptualización del trabajo en la vejez es imprescindible tender un puente entre la dimensión micro y macro que permita trasladar los significados, experiencias y valores a los contextos históricos, políticos y culturales que se construyen en esta era globalizada. Finalmente, el poder definir con mayor precisión lo que es el trabajo en la vejez va a permitir visibilizar con claridad la condición multidimensional de este fenómeno.

Se considera que, a través de la teoría fundamentada, se puede consolidar este vínculo entre la conceptualización, la teorización y el acercamiento con la realidad del trabajo en la vejez, para crear una teoría sustantiva que emerge de la propia voz de las personas mayores y que permita realizar un análisis más profundo de lo micro y lo macrosocial. En salud pública este tipo de investigaciones contribuye a ir más allá del dato duro y del acercamiento meramente descriptivo, dando pie a la generación de análisis críticos de la realidad y ayuda a la capacidad de producir información que permita construir propuestas de intervención más eficaces y eficientes con este sector de la población.

## Consideraciones finales

El análisis crítico realizado en el presente ensayo sobre las perspectivas utilizadas para conceptualizar el trabajo en la vejez permitió distinguir que existen dos niveles de aproximación: micro y macrosocial. En cada nivel se desarrollan conceptos que integran elementos teóricos y metodológicos asociados a paradigmas (explicativo, interpretativo y crítico), así como al desarrollo de marcos de política pública y derechos humanos.

En el nivel microsocial se reconoce la relevancia de las experiencias laborales en las trayectorias de vida y las transiciones de roles que se producen en la vejez, consolidando una diversidad de maneras de envejecer y de trabajar. Comprender que existen condiciones individuales, familiares, sociales y estructurales que limitan o permiten que las personas mayores continúen trabajando, contribuye a la posibilidad de conceptualizar y posteriormente teorizar sobre este fenómeno.

A su vez, en el nivel macrosocial se reconoce que el aspecto económico tiene un peso específico en el tema laboral, puesto que es un eje central que determina las decisiones que toman las organizaciones y los estados sobre el desarrollo del mercado laboral y las maneras de regularlo. Por su parte, los derechos humanos son la guía que permite establecer un punto de equilibrio en las relaciones laborales y pueden ser un freno para el capitalismo salvaje y la explotación laboral. Mientras que la perspectiva de género ha puesto en el ojo del huracán las desigualdades que viven las mujeres bajo el modelo sexo/género y como son trazadas mediante la división sexual del trabajo. 

El estudio del trabajo en la vejez es aún limitado, las investigaciones se mantienen en un nivel descriptivo, por ende, las conceptualizaciones que se construyen todavía no logran consolidar una teoría general a nivel explicativo que permita establecer una definición del concepto: trabajo en la vejez.

La conceptualización diferenciada en dos niveles de interacción, micro y macrosocial es insuficiente para explicar las dinámicas que surgen en el trabajo durante la vejez porque carecen de un proceso de vinculación (nivel meso) que permita integrar los elementos objetivos y subjetivos con el contexto en el que se desarrollan las personas mayores.

Finalmente, este análisis crítico ofrece la posibilidad de comenzar un debate más profundo sobre el trabajo en la vejez, sus implicaciones económicas, sociales y políticas. Este estudio presenta una limitación de alcance, puesto que solo se analizaron las conceptualizaciones propuestas en Europa y América. Este ensayo contribuye a poner en la mesa el tema del trabajo en la vejez, para darnos cuenta de que debemos pasar de la conceptualización a la definición, porque lo que no se puede nombrar, no se puede ver. Aporta a la salud pública la posibilidad de reconocer las variables que están en juego en este problema que afecta a un grupo de población en crecimiento y que requiere de un abordaje transdiciplinario.

## Data Availability

Las fuentes de información utilizadas en el estudio se indican en el cuerpo del artículo.

## References

[1] Katz H, Wailes N, Hernández M (2014). Los nuevos estudios laborales en México: perspectivas actuales..

[2] Ramos G, Tirado E (2019). Hasta que el cuerpo aguante trayectorias, rutinas y motivaciones laborales de trabajadores adultos mayores de la ciudad de Lima, Perú. Bull Inst Fr Études Andines.

[3] Dingemans E, Henkens K, van Solinge H (2017). Working retirees in Europe individual and societal determinants. Work Employ Soc.

[4] Comisión Económica para América Latina y el Caribe (2022). Envejecimiento en América Latina y el Caribe: inclusión y derechos de las personas mayores.

[5] Comisión Europea (2021). Libro verde sobre el envejecimiento: fomentar la solidaridad y la responsabilidad entre generaciones..

[6] Marafioti R (2003). Los patrones de la argumentación: la argumentación en los clásicos y en el siglo XX.

[7] Lytle MC, Foley PF, Cotter EW (2015). Career and retirement theories relevance for older workers across cultures. J Career Dev.

[8] Robledo CA, Orejuela JJ (2020). Teorías de la sociología, el envejecimiento y la vejez. Revista Guillermo de Ockham.

[9] Mirelles I (2011). Envejecimiento productivo las contribuciones de las personas mayores desde la cotidianidad. Trabajo y Sociedad.

[10] Waddell C, Van Doorn G, Power G, Statham D (2024). From successful ageing to ageing well a narrative review. Gerontologist.

[11] Organización Mundial de la Salud (2002). Envejecimiento activo un marco político. Rev Esp Geriatr Gerontol.

[12] George LK (1993). Sociological perspectives on life transitions. Annu Rev Sociol.

[13] Afonso M (2012). Las construcciones identitarias en el trabajo en la contemporaneidad retrato de un grupo de trabajadores de São Paulo (Brasil). Psykhe (Santiago).

[14] Comisión Económica para América Latina y el Caribe Organización Internacional del Trabajo (2018). La inserción laboral de las personas mayores: necesidades y opciones.

[15] Brown Grossman F, Nava Bolaños I (2024). Transiciones laborales de las personas mayores en México. Rev Mex Sociol.

[16] Muñoz CG, Reinoso Fica LA, Cirineu CT, Pizarro Troncoso E (2024). El trabajo en la vejez un desafío para la terapia ocupacional y los estudios sobre la ocupación. Rev Ocup Hum.

[17] Eagers J, Franklin RC, Broome K, Yau MK (2019). The experiences of work retirees' perspectives and the relationship to the role of occupational therapy in the work-to-retirement transition process. Work.

[18] Baxter S, Blank L, Cantrell A, Goyder E (2021). Is working in later life good for your health A systematic review of health outcomes resulting from extended working lives. BMC Public Health.

[19] Malinowska D, Tokarz A, Wardzichowska A (2018). Job autonomy in relation to work engagement and workaholism mediation of autonomous and controlled work motivation. Int J Occup Med Environ Health.

[20] De la Garza E, De la Garza E, Hernández M (2020). Configuraciones productivas y circulatorias y trabajo no clásico en los servicios..

[21] Vargas E, De la Garza E, Hernández M (2020). Configuraciones productivas y circulatorias y trabajo no clásico en los servicios..

[22] Sánchez M, Stampini M, Ibarrarán P, Vivanco F, Castillo P, Buenadicha C (2020). La economía plateada en América Latina y el Caribe: el envejecimiento como oportunidad para la innovación, el emprendimiento y la inclusión.

[23] Casas JI (2024). Una visión crítica de la economía plateada. Dossieres EsF.

[24] Blustein DL, Olle C, Connors-Kellgren A, Diamonti AJ (2016). Decent work a psychological perspective. Front Psychol.

[25] Ghai D (2003). Trabajo decente, concepto e indicadores. Revista Internacional del Trabajo.

[26] Cuéllar E, Huitrón MG, Tapia W (2023). Pensiones: ¿qué podemos aprender de países con procesos de envejecimiento más avanzados?. Factor Trabajo.

[27] Pineda R, Albornos S, Aravena C, Gálvez T (2024). Empleo informal en América Latina: grupos más propensos..

[28] Rubin G, Lamas MC (1997). El género: la construcción cultural de la diferencia sexual..

[29] Bustos B (2011). Familia y trabajo en la Zona Metropolitana de Guadalajara. División sexual del trabajo a finales del siglo XX.

[30] Moore K, Ghilarducci T (2018). Intersectionality and stratification in the labor market. J Am Soc Aging.

[31] Arreseigor M, Martínez GM (2023). Masculinidad(es) en la vejez la cara oculta del género. Pensamiento y Acción Interdisciplinaria.

[32] Blanco M, Pacheco E (2003). Trabajo y familia desde el enfoque del curso de vida dos subcohortes de mujeres mexicanas. Papeles de Población.

[33] Oddone MA, Chernobilsky L (2019). Envejecimiento productivo el trabajo después de los sesenta. Rev Argent Gerontol Geriatr.

[34] Fuentes-García A (2025). Trabajo, jubilación y vejez: paradojas del trabajo como derecho.

[35] Porcellato L, Carmichael F, Hulme C, Ingham B, Prashar A (2010). Giving older workers a voice constraints on the employment of older people in North West England. Work Employ Soc.

[36] Lóyzaga O (2017). El derecho al trabajo. Un análisis crítico..

[37] Somavía J (2014). El trabajo decente. Una lucha por la dignidad humana.

[38] Gardiner J, Eisenstein Z (1980). Patriarcado capitalista y feminismo socialista.

[39] Viveros M (2023). Interseccionalidad, giro decolonial y comunitario.

[40] Connell RW, Messerschmidt WJ (2005). Hegemonic masculinity rethinking the concept. Gend Soc.

[41] Atchley R (1989). A continuity theory of normal aging. Gerontologist.

[42] Giele J, Elder G (1998). Methods of life course research. Qualitative and quantitative approaches.

[43] Deci EL, Ryan RM (1985). Intrinsic motivation and self-determination in human behavior.

[44] Naciones Unidas (2015). Declaración universal de los derechos humanos..

[45] Scott JW, Lamas MC (2013). El género: la construcción cultural de la diferencia sexual..

[46] Einsenstein Z, Einsenstein Z (1980). Patriarcado capitalista y feminismo socialista..

